# Obesity Is an Independent Predictor of Poor Survival in Metastatic Breast Cancer: Retrospective Analysis of a Patient Cohort Whose Treatment Included High-Dose Chemotherapy and Autologous Stem Cell Support

**DOI:** 10.4061/2011/523276

**Published:** 2011-07-06

**Authors:** A. von Drygalski, T. B. Tran, K. Messer, M. Pu, S. Corringham, C. Nelson, E. D. Ball

**Affiliations:** ^1^Division of Hematology/Oncology, Department of Medicine, UCSD, CA 92093, USA; ^2^Moores Cancer Center, 3855 Health Sciences Drive, La Jolla, Ca 92093-0829, USA; ^3^Division of Biostatistics and Bioinformatics, Department of Family and Preventive Medicine, UCSD, CA 92093, USA; ^4^Division of Bone and Marrow Transplantation, Department of Medicine, UCSD, CA 92093, USA

## Abstract

The purpose of the study was to identify predictors of long-term survival in metastatic breast cancer (MBC). A cohort of 96 patients, who received high-dose chemotherapy with autologous stem cell support (HD-ASCT) as part of their treatment, was analyzed. Percent long-term survival at 10 years was 24.5% (CI 17.2–34.9%) when metastasis was diagnosed and 14.4% (CI 8.7–23.9%) when MBC was diagnosed. Survival was impacted significantly by body mass index (BMI). Median overall survival from initial diagnosis or from time of metastasis for patients with BMIs ≤30 and >30 (obese) was 7.1 (CI 4.4–8.7) and 3.2 years (2.41–6.75), respectively, or 3.2 or 2.3 years (all *P* = 0.02). Also, obesity was the only independent patient-related predictor of time to metastasis and of survival. While obesity is linked with poor outcomes in earlier stages of breast cancer, this has not been previously reported for MBC.

## 1. Introduction

Breast cancer is the second leading cause of cancer deaths in women today after lung cancer. Metastatic breast cancer (MBC) is deemed incurable and median survival for patients with cancer that is estrogen receptor (ER) negative or no longer hormonally responsive is only 18 to 24 months [1, 2].

A variety of molecularly targeted drugs have been developed for MBC, but to date only, trastuzumab (Herceptin, Genentech, CA, USA) has shown an overall survival (OS) benefit of several months [3–5]. Clearly, more effective treatment strategies and/or improved patient selection are urgently needed. In the 1990s, high dose chemotherapy followed by autologous stem cell transplantation (HD-ASCT) was pioneered to improve survival. The initial feasibility studies in small cohorts of patients with advanced stage disease showed improved survival when compared to historical controls [6–9]. Subsequent prospective randomized clinical trials comparing HD-ASCT to standard chemotherapy resulted in improved PFS, but failed to demonstrate OS benefit [10–14]. The results of the last trial of these series with 386 patients with metastatic breast cancer employing HD-ASCT were reported most recently and confirmed the results of earlier studies [15]. All together, the lack of convincing survival data at the expense of high toxicity resulted in the demise of HD-ASCT as a treatment modality of MBC.

However, while the vast majority of patients with MBC will succumb to their disease within a relatively short period of time, 5–10% of patients live longer than 5 years, and observational data suggest that 1–3% of patients treated with conventional chemotherapy and/or hormonal manipulation may experience long-term survival beyond 10–15 years [16–21]. Quite surprisingly, the most impressive long-term survival rates in excess of 10% were reported with HD-ASCT [22–24]. Response to treatment, site of metastases, hormone receptor status, performance status, and short initial disease-free interval were important prognostic factors for survival after HD-ASCT and corresponded to prognostic factors known from treatment with conventional, less aggressive therapies [25–27]. However, the observation periods in all those studies rarely exceeded 5 years. 

We have studied long-term survival rates as well as the distinct disease- and patient-related characteristics that would be predictive of long-term survival in a cohort of patients with MBC, who were treated at our institution between 1989 and 1999. Treatments for all patients were not limited to, but included HD-ASCT.

## 2. Methods

### 2.1. Patient Population and Data Extracted

Records of all patients in the bone marrow transplant registry at UCSD treated with HD-ASCT for MBC between 1989 and 1999 were retrospectively reviewed. All patients were females. Patients were followed for a median of 65 months from diagnosis (range 10.4–255.0 months; quartiles 36.7–109.3 months). No patient was excluded from analysis. Data acquisition and patient confidentiality safeguards were approved by the Institutional Review Board. Age, race, stage at diagnosis, histology, estrogen receptor (ER) and menopausal status, body mass index (BMI) in kg/m^2^ at the time of HD-ASCT, time to transplant and death, site of metastasis, and disease status prior to HD-ASCT were extracted. Since all transplants were carried out prior to routine assessment of Her-2/neu receptor status, this information was not part of our analysis. A BMI > 30 was defined as obesity (http://www.cdc.gov/). Menopause was defined clinically by absence of menstruation for more than 12 months (http://www.cancer.gov/).

### 2.2. Treatments

High dose chemotherapy prior to stem cell support was platinum based in combination with thiotepa/etoposide, ifosfamide/etoposide, cyclophosphamide/BCNU, cyclophosphamide/mitoxantrone, or cyclophosphamide/thiotepa. Dosing was strictly weight-based. Body surface area was allowed to exceed 2 m^2^. Ten patients received HD-ASCT in first line; all other patients received at least one line of treatment prior to HD-ASCT (median 1; range 0–4). Hormonal treatments for ER+ patients were administered at physicians' discretion. Autologous stem cell rescue was performed with harvested bone marrow cells in 6 patients. The other 89 patients received peripherally mobilized stem cells, 23 of which received grafts supplemented with harvested bone marrow cells. Mobilization was achieved with granulocyte- or granulocyte/macrophage-colony stimulating factor with or without cyclophosphamide.

### 2.3. Assessment of Disease Status

Extent of metastatic disease was determined within 12 weeks prior to and within 6–12 weeks after HD-ASCT by imaging and physical exam. Thereafter, assessment of disease status was prompted by patient symptoms and carried out at physicians' discretion. Patients were grouped according to site of metastasis : in visceral or bone metastasis, or local or nodal recurrence. If multiple sites of metastasis were present, assignment to one metastatic site was prioritized as follows: visceral > bone > nodal > local. Complete response (CR), partial response (PR), stable disease (SD), and progressive disease (PD) were determined according to RECIST criteria.

### 2.4. Statistical Analysis

Multiple linear regression models were used to explore the association between a continuous outcome and predictors of interest. Residual analysis was performed to check for outliers, normality assumption, and heteroscedasticity. Transformation was done on the outcomes if necessary. Brookmeyer and Crowley's 95% confidence intervals were used for median survival times [28]. PFS and OS were estimated using Kaplan-Meier method. Cox models were used to identify independent predictors of a time-to-event variable and Schoenfeld tests were used to test for proportional hazard assumptions. Predictors of interest with *P* < 0.2 in the univariate analysis were included in an initial model and a manual backward selection was made based on the Wald test *P* values. One subject with an unknown stage value was excluded from the final models. All analyses used the statistical package R version 2.5.1, 2007. (http://www.r-project.org/).

## 3. Results

### 3.1. Patient and Disease Characteristics

Patient-related characteristics are listed in Table 1. Median age at initial diagnosis was 43 years (quartiles 38–49) and 47 years (quartiles 41–43) at metastasis. Median time from initial diagnosis to documentation of metastasis was 29 months (quartiles 16.6–63.7), and from metastasis to HD-ASCT 6.6 months (quartiles 4.5–16.6), respectively. Twelve patients received HD-ASCT as their first line of treatment, whereas 84 patients had at least one prior line of chemotherapy for metastatic disease (48 patients with 1 line, 24 with 2 lines, 10 patients with 3 lines, and 2 patients with 4 lines of chemotherapy). Median BMI at metastasis was 25 (quartiles 21.4–29.6) and 24% of patients were obese (BMI > 30).

### 3.2. Survival Rates and Influence of BMI on Survival

**Figure 1:**
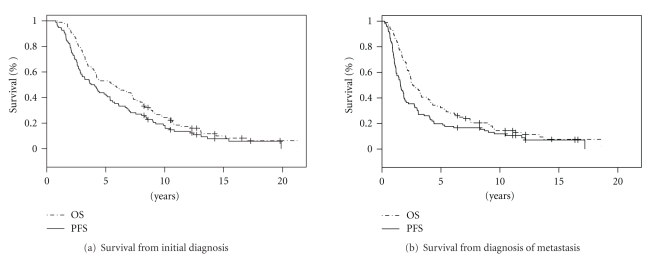
Overall survival and progression-free survival for all patients (*n* = 96) from initial diagnosis (a) and diagnosis of metastasis (b). Survival was estimated by Kaplan-Meier method.

**Figure 2:**
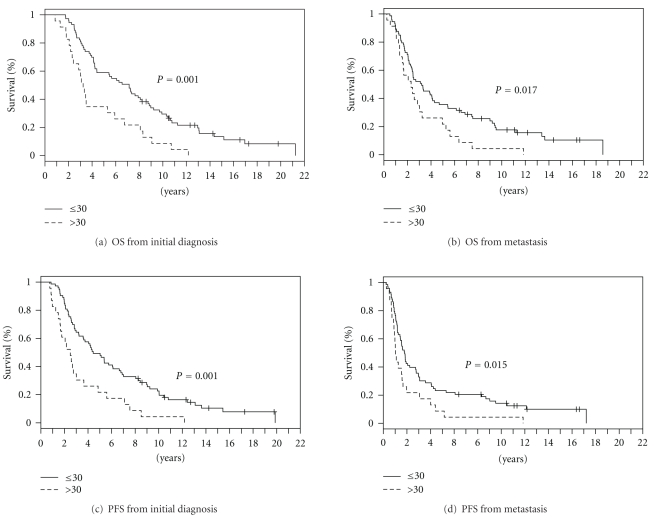
Overall survival (OS) from diagnosis (a) and from time of metastasis (b) and progression-free survival (PFS) from diagnosis (c) and from time to metastasis (d) in patients with a BMI ≤ 30 (*n* = 73) or >30 (*n* = 23). Survival was estimated using Kaplan-Meier method.

For all patients (*n* = 96), median PFS and OS were 3.9 years (CI 2.9–5.4) and 5.6 years (CI 4.1–7.4) after initial diagnosis, and 1.6 years (CI 1.3–1.9) and 2.7 years (CI 2.3–3.9) after diagnosis of metastatic disease, respectively (Figures 1(a) and 1(b)). Survival rates at 10 years were 24.5% (CI 17.2–34.9%) from diagnosis and 14.4% (CI 8.7, 23.9%) from when metastatic disease was diagnosed. As depicted in Figure 2, PFS and OS differed significantly for patients with a BMI ≤ 30 and >30. At diagnosis, median PFS for patients with BMIs ≤ 30 (*n* = 73) and >30 (*n* = 23) was 4.4 (CI 3.6–6.7) and 2.5 years (CI 1.7–4.9), and median OS was 7.1 (CI 4.4–8.7) and 3.2 years (2.41–6.75), respectively (Figure 2(a) and 2(c); all *P* values = 0.001; univariate). Once metastasis was diagnosed, median PFS for patients with BMIs ≤ 30 and >30 was 1.8 (CI 1.44–2.77) and 1.04 years (0.9–1.96) years, and median OS was 3.20 (CI 2.42–4.30) and 2.30 years (CI 1.56–4.96), respectively (Figure 2(b) and 2(d); all *P* values <0.02; univariate).

### 3.3. Independent Predictors of Time from Initial Diagnosis to Metastasis

Ethnicity, stage at diagnosis, histology, site of metastasis, remission prior to HD-ASCT, menopausal status, ages at HD-ASCT and at diagnosis (< age 40 versus ≥ age 40), BMI (≤ 30 versus > 30), and ER status were evaluated as independent predictors of PFS and OS. In a univariate analysis, BMI, stage at initial diagnosis, and histology were predictive of time from initial diagnosis to metastatic disease (all *P* values <0.01). As depicted in Table 2, only the initial stage at diagnosis and BMI were confirmed as independent predictors in a multivariate multilinear regression analysis. Time to metastasis was significantly shorter for patients with a BMI > 30 (Table 2). Median time to metastasis was 1.14 years for patients with a BMI > 30 compared to 1.85 years for patients with a BMI ≤ 30 (*P* < 0.04 for time to metastasis). Times to metastasis were similar for stage I or II disease (approximately 2 years), but time to metastasis shortened significantly compared to stage I or II disease when stage III was present at diagnosis (0.72 years, *P* < 0.0006).

### 3.4. Independent Predictors of Survival from Metastasis

Stage at diagnosis, ER-status, site of metastasis, remission status prior to HD-ASCT, and BMI were considered for multivariate analysis since univariate *P* values were <0.02. Only BMI, stage at diagnosis and site of metastasis were confirmed to be independent predictors of survival at the time of metastasis (Table 3). Patients with a BMI > 30 had a significantly higher hazard of progression and death compared to patients with a BMI ≤ 30 (HR for progression = 2.23; *P* = 0.005; HR for death  =  1.82; *P* = 0.04). Also, once metastasis was diagnosed, hazards of progression and death increased significantly with increasing stages present at diagnosis (compared to stage I all *P* values <0.05; Table 3) or with visceral disease.

## 4. Discussion

In the 1990s, high HD-ASCT was thought to be a beneficial treatment option in MBC, based on the hypothesis that significant dose escalation would improve the efficacy of chemotherapy. When several randomized trials demonstrated that OS was not significantly improved [1–15]. HD-ASCT was abandoned. Since cure could not be achieved with aggressive treatment, the school of thought changed and sequential single agent chemotherapy with or without biological targeted therapies and /or hormonal manipulation in ER+ patients has become the preferred way of symptom control [2, 29]. 

However, a proportion of patients with MBC can achieve long-term survival which has been reported to exceed 10% with more aggressive chemotherapy such as HD-ASCT [22–24]. We were specifically interested if such high percentages of survival could be maintained beyond 10 years, at which point it is possible that at least some of these patients may be cured of their disease. Second, we studied the patient- and disease-specific characteristics predictive of survival. For both questions we analyzed our institutional cohort of 96 patients with MBC who had received HD-ASCT as part of their treatment algorithm. Since the last patient was transplanted in 1999, each patient had a minimum follow-up of 10 years. 

First, we did find long-term survival in excess of 10 years. Survival rates at 10 years were 24.5% from initial diagnosis and 14.4% from when metastatic disease was diagnosed. 

Second, and most surprisingly, in univariate and multivariate analyses, obesity was the only patient-related factor associated with significantly shorter OS and PFS from initial diagnosis and from when metastasis was diagnosed. Obesity also predicted shorter time from diagnosis to metastasis. Obesity has previously been reported as a risk factor for survival in patients diagnosed with earlier stages of breast cancer [30–32] and seems associated with higher stages at diagnosis [33], but, to the best of our knowledge, this is the first study reporting obesity as a risk factor for survival in patients with MBC. Other patient-related factors such as age, ethnicity, and menopausal status were not significant. Weights and obesity status were not available to us at the time of initial diagnosis, but recorded at the time of metastasis prior to first treatment. The median interval between initial diagnosis and time to metastasis was only approximately 3 years and can probably be considered a time frame where major weight changes are not expected to occur for the majority of patients [34]. We therefore felt comfortable to use the weight obtained at metastasis as the representative personal weight for each patient. 

Third, among disease-related characteristics, stage at diagnosis was an independent predictor of time to metastasis. Histology and ER-status were not predictive in our cohort. Her-2/ neu was not part of the analysis since it was not routinely tested prior to 1999. At the time of metastasis, in addition to stage at diagnosis, visceral metastasis also became an independent predictor of a higher hazard for progression and, suggestively, shortened OS. Higher stages at diagnosis are known to be associated with shorter time to recurrence and poorer survival [35, 36], and increased likelihood of occult metastasis in permissive niches such as the bone marrow and/or biologically more aggressive forms of cancer with increased burden of disease were suggested as plausible explanations for poorer survival at higher stages [37, 38]. Also, visceral metastasis has been reported before as a predictor of poor survival in MBC [25–27]. The association of poorer outcomes with higher stages at initial diagnosis or visceral metastasis seems uniformly accepted, and our study supports these previous findings. The most important and novel finding was the negative effect of obesity on survival in MBC, and its importance as independent predictor of time to metastasis and progression. To the best of our knowledge, these results provide first strong evidence that elevated BMI may contribute significantly to poor survival in MBC and these data were ascertained in the setting of HD-ASCT. While it has been reported that obesity is associated with higher stages of breast cancer at diagnosis [33], and that obesity negatively impacts various outcome factors in earlier stages of breast cancer [30–32], the effects of obesity have not been directly studied in MBC. Specifically in respect to locoregional breast cancer, two prospective studies identified obesity as an independent risk factor for overall mortality, distant and contralateral recurrence, and death from breast cancer [31, 32]. Similar findings for all presentations of breast cancer (some of which had MBC) were reported in the Prospective Analysis of Case-control studies on Environmental factors and Health (PACE) study [30].

In general, it is increasingly recognized that obesity and cancer are linked and that obesity not only confers a higher risk of cancer incidence but also increases cancer mortality as demonstrated in three large population-based cohort studies [39–41]**. **The reasons are poorly understood, but tumor growth stimulation by adipokines released from fatty tissue, inflammation [42–44], and especially in the case of breast cancer increased estrogen levels in obese postmenopausal females fueling tumor growth [45, 46] are implicated. Also, adjuvant undertreatment of obese patients, if chemotherapy is not strictly weight-based [47–49], is increasingly recognized as important for early relapse. Against physicians' beliefs, chemotherapy in the obese is well tolerated, and, while dose increases proportional to weight do not confer a higher risk for toxicity [49, 50], under-dosing in the obese breast cancer patient receiving adjuvant chemotherapy demonstrated increased risk for recurrence, shorter DFS or OS [51, 52]. Despite these data, two recent surveys show that only 25% of breast cancer patients receive strictly weight-based dosing during adjuvant chemotherapy and that 19 of 44 cooperative group trials used dose limits for the obese [47, 48]. In our study population, doses of previous adjuvant regimens were unknown, and suboptimal adjuvant treatment cannot be ruled out as a reason for faster time to metastasis in the obese. However, agents used for treatment in the metastatic setting and for HD-ASCT were dosed by actual body weight, and body surface areas were allowed to exceed 2 m^2^, such that underdosing does not appear to be the main reason for poorer outcome parameters. Further study limitations are absence of Her-2-neu as important prognostic marker, since it was not routinely available in the era of HD-ASCT, and patient population diversity. Selection bias may be present since not every patient deemed a candidate for HD-ASCT may have been referred or proceeded to HD-ASCT. Factors such as, but not limited to, physicians' or patients' treatment preferences, patients' performance status and socioeconomic status may have interfered. 

Until now, obesity and its effect on outcomes in MBC have not been examined in depth, and this is the first report to implicate obesity in poorer outcomes. While this observation was made in the setting of HD-ASCT, conclusions about the contribution of HD-ASCT to long-term survivorship cannot be made, given retrospective study design, diverse study patient population, and mixed treatment algorithms. However, our results demonstrate that ultra-long-term survivorship can be achieved in the setting of more aggressive chemotherapy with HD-ASCT and that obesity was deleterious in MBC. In contrast to MBC, correction of unhealthy life-style behavior associated with obesity is the subject of intense exploration after adjuvant treatment for breast cancer, where patients are officially recognized as “breast cancer survivors.” Results from the Women's Healthy Eating and Living (WHEL) study [53] suggest that obese patients can experience significant overall survival benefit if compliant with a combination of diet and exercise. We believe that our findings generate the hypothesis that obesity may be a potentially correctable patient-related adverse survival factor in MBC and, similar to approaches for locoregional stages of breast cancer, can serve as a target for intervention given the prospect of long-term survival in certain settings.

## Figures and Tables

**Table 1:** Patient and disease characteristics.

	*N *
Race	
White	81
Hispanic	7
Asian	4
African-American	4

Age at metastasis	
<age 40	19
≥age 40	77

Menopausal status at diagnosis	
Premenopausal	28
Postmenopausal	68
Unknown	2

BMI at metastasis	
<30	73
≥30	23

Stage at diagnosis	
Stage I	21
Stage II	43
Stage III	23
Stage IV	8
Unknown	1

Histology	
Infiltrating ductal	79
Infiltrating lobular	10
Unknown	7

Estrogen receptor status	
Positive	57
Negative	36
Unknown	3

Site of metastasis	
Visceral	38
Bone	28
Lymph nodes	14
Local	16

**Table 2:** Independent risk factors for time from initial diagnosis to metastasis.

		Years from initial diagnosis to metastasis		
		Median (*Q*1, *Q*3)	Multiple linear model*	
			Coefficients (95% CI)	*P*
	*n*		Intercept : 0.87 (0.48, 1.26 )	

BMI				
<30	73	1.85 (0.97, 3.26 )	Ref	
≥30	23	1.14 (0.32, 1.97 )	−0.44 (−0.85, −0.02 )	0.04
Stage at diagnosis				
I	21	2.06 (1.37, 3.26 )	Ref	
II	43	2.01 (1.41, 3.29 )	0.02 (−0.44, 0.48)	0.92
III	23	0.72 (0.19, 1.94 )	−0.93 (−1.46, −0.41)	0.0006
IV ( = metastasis)	8	N/A	N/A	N/A

*log⁡(years from initial diagnosis to metastasis + 0.1) was used as the outcome.

**Table 3:** Independent risk factors for progression-free survival (PFS) and overall survival (OS) at diagnosis of metastasis.

	*n*	PFS		OS	
		HR (95%CI)		HR (95% CI)	
		HR (95% CI)	*P*	HR (95% CI)	*P*
BMI					
<30	72	1		1	
≥30	23	2.23 (1.28, 3.88)	0.005	1.82 (1.03, 3.23)	0.04
Stage at diagnosis					
Stage I	21	1		1	
Stage II	43	5.09 (2.55, 10.16)	<0.001	2.87 (1.53, 5.4)	0.001
Stage III	23	3.58 (1.61, 7.94)	0.002	2.61 (1.22, 5.58)	0.01
Stage IV	8	6.88 (2.54, 18.6)	<0.001	3.88 (1.52, 9.9)	0.04
Site of metastasis					
Local	16	1		1	
Nodal	14	1.37 (0.59, 3.2)	0.47	1.38 (0.6, 3.16)	0.45
Bone	28	1.79 (0.84, 3.82)	0.13	1.60 (0.76, 3.38)	0.22
Visceral	37	4.45 (2.12, 9.37)	<0.001	2.54 (1.25, 5.15)	0.01
